# Method for an Effective Selection of Tools and Cutting Conditions during Precise Turning of Non-Alloy Quality Steel C45

**DOI:** 10.3390/ma15020505

**Published:** 2022-01-10

**Authors:** Oleksandr Ivchenko, Vitalii Ivanov, Justyna Trojanowska, Dmytro Zhyhylii, Olaf Ciszak, Olha Zaloha, Ivan Pavlenko, Dmytro Hladyshev

**Affiliations:** 1 Department of Manufacturing Engineering, Machines and Tools, Sumy State University, 2, Rymskogo-Korsakova Str., 40007 Sumy, Ukraine; o.ivchenko@tmvi.sumdu.edu.ua (O.I.); zaloga.o@tmvi.sumdu.edu.ua (O.Z.); d.hladyshev@tmvi.sumdu.edu.ua (D.H.); 2 Faculty of Mechanical Engineering, Poznan University of Technology, Piotrowo 3, 60-965 Poznan, Poland; justyna.trojanowska@put.poznan.pl (J.T.); olaf.ciszak@put.poznan.pl (O.C.); 3 Department of Computational Mechanics Named after Volodymyr Martsynkovskyy, Sumy State University, 2, Rymskogo-Korsakova Str., 40007 Sumy, Ukraine; d.zhigiliy@omim.sumdu.edu.ua (D.Z.); i.pavlenko@omdm.sumdu.edu.ua (I.P.)

**Keywords:** edge cutting tool, finite element model, precision machining, cutting process, industrial growth, simulation design, optimization function, friction coefficient

## Abstract

The paper presents a constructing methodology for a modern approach to tools selection and solving the problem of assigning optimal cutting parameters for specific production conditions. The mathematical formulation determining the extreme values of the technological process optimality criteria is obtained. A system of technical and economic quality indicators for cutting tools is proposed. This system allows principles’ implementation of decentralization and interoperability “Industry 4.0” via finite element modeling of the cutting process based on solving the problem of orthogonal free cutting modeling. The proposed methodology further usage is possible by creating a standardized database on the parameters of the tool: the adhesive component of the friction cutting coefficient for processing of a specific pair of cutting and tool materials (or tool coating material) and the impacts of the cutting-edge radius on cutting efficiency of a particular material.

## 1. Introduction

At present, the world is on the verge of the fourth industrial revolution, Industry 4.0, related to the “Internet of things” (IoT) production [1], which involves using the computer networks of physical objects, interaction with unique or virtual identifiers [2] and data exchange between all production system components allowance, as well as with the external environment [3].

**Figure 1:**
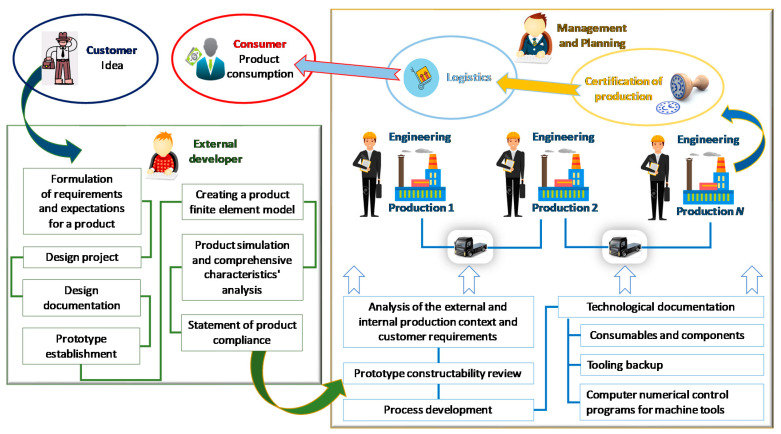
A modern conditional model of the concept implementation of introducing new non-food products from idea to final product.

Figure 1 shows a modern conditional model of introducing new non-food products from idea to final product. This model has several drawbacks in applying IoT, particularly engineering processes to link to the production process (block “Management and Planning”). It does not allow full implementation of some “Industry 4.0” principles [4]. The first one is interoperability as the inability to communicate using IoT between a cyber-physical system (e.g., parts’ carriers, processing assembly stations), blocks “External developer” or “Customer” and “smart” manufacturing. The second principle is decentralization as a cyber-physical system’s inability to make personal decisions within the “smart” industries framework.

One of the solutions is the virtualization of “smart” productions [5]—a digital double, a virtual simulation model of production. This problem has virtually been solved for some applied production technologies (3D printing) [6]. Simultaneously, the mainstream technology for machining specific manufacturing engineering products remains the cutting-edge machining of materials [7].

An essential role in developing Industry 4.0 for ensuring material machining plays the following previous research. The design of innovative surface technology for smart cutting tools during high-value machining was proposed in 2014 by C. Wang et al. [8]. In 2016, B. Denkena et al. [9] studied a novel continuous generating grinding process to produce cutting tools. Additionally, in 2020, V. Kalchenko et al. [10] carried out a three-dimensional simulation of the precision machining on a CNC machine tool.

In 2017, S. Klimenko et al. [11] studied contact stresses on the rake face of cutting tools during materials machining. In 2019, I. Karabegovic et al. [12] developed the ways of using smart sensors in production processes according to Industry 4.0. According to the IoT trend, another innovative control technology for machine tools was developed in 2016 by M. Fujishima et al. [13].

In 2018, F. Silva et al. [14] described Cloud Computing environments for simulation of adaptable systems in Industry 4.0. In 2019, S. Saniuk et al. [15] proposed using Cyber Industry Networks as an Industry 4.0 implementation environment.

The investigations of the surface layer properties of materials (e.g., for the DIN C45 Steel case study) after cutting were performed in 2020 by K. Zaleski et al. [16]. Additionally, R. Maruda, S. Legutko et al. [17] applied the Parameter Space Investigation Method to determine cutting power under minimized cooling conditions during the material machining for the same case study.

In 2019, S. Dobrotvorsliy et al. [18] proposed the concept of the software for materials selection using .NET Technologies. Wireless Sensor Network’s implementation for the advancement in IoT using data validation algorithms and artificial intelligence techniques was later realized in 2020 by T. Kanwal et al. [19]. Finally, ways to improve the quality of cutting tools state recognition using cloud technologies were proposed in 2020–2021 by Fomin and Derevianchenko [20,21]. Paper [22] focuses on cutting process modeling, correlating, and optimizing the critical process parameters using the Taguchi method under the AISI P20 milling operation to reduce surface roughness. The statistical analysis of the numerical and physical experiments made it possible to develop a mathematical model and optimum solutions for assessing surface roughness under the milling process.

In 2021, Xu L.-H. et al. [23] proposed a new adaptive neuro-fuzzy inference system (NANFIS) for power consumption and surface quality predictions. The NANFIS model obtains the cutting parameters as inputs and outputs the machining performances. The proposed intelligent system is implemented into the high-speed milling process of compacted graphite iron. The experimental results show that adopting the NANFIS models predicts energy consumption and surface roughness to the accuracy within 91.2% and 93.4%, respectively.

In [24], a system for surface defects classification made under milling CRF/PEEK was suggested for the prediction model of surface quality considering fiber orientation, cutting speed, feed per tooth, and cutting width using a neural network optimized for the genetic algorithm (GA-BP). The results’ prediction shows that the model has sufficient generalization capability with a prediction accuracy above 90.39%.

This article’s study object was the parts finishing, usually including edge cutting machining, which determines the machined surface’s geometric dimensions, shape, and quality (surface layer). Since the fact that one of the primary finishing machining features is a relatively small depth of cut (from 0.1 to 0.4 mm) and feed and, accordingly, small chip thickness and relatively high tool-cutting speeds, special requirements for sharpness are imposed on the cutting tool edges and the actual friction coefficient value under the cutting process. Notably, the tool edge radius should be sufficiently small, which means not exceeding the established limits. The friction coefficient on the cutting blade (especially its adhesive component) substantially depends on the contact conditions between the blade and chips and surfaces on the workpiece, i.e., cutting conditions. Meanwhile, the process should not be accompanied by a significant cutting temperature and an actual blade wear rate.

An investigation into recommendations of well-known manufacturers of edge cutting tools (*ECT*) on the machining modes choice establishes the presence of special software systems for calculation or reference data (catalogs). At present, manufacturers have a huge experience in choosing proper cutting tools for different types of materials. However, an essential disadvantage is a quite wide range of cutting modes. Therefore, wide dispersion of cutting forces and torques can occur. This fact leads to different machining efficiency within the same types of the recommended insert. Finally, there is an urgent problem to develop an effective approach to substantiate the cutting tool’s selection by ensuring a narrower range of parameters corresponding to the principles of Industry 4.0.

Therefore, the creation methodology for applying material machining processes virtualization in *ECT* will move one major step closer to implementing IoT as a critical link to ensure the rapid introduction of new non-food products from idea to a final product.

The article aims to virtualize material machining (e.g., precision machining of *ECT*) by developing a general selecting *ECT* model to ensure optimal cutting conditions by using simulation and developing the technical and economic indicators system of the quality of the cutting tools.

However, the research mentioned above does not entirely realize a generalized model of the edge cutting tools choice to ensure optimal cutting conditions.

The paper is based on the research of the contact zone of “tool–processed part” formation process under the adhesion conditions, and the dependence of the turning inserts quality on an adhesive component of the friction force is established. The novelty of the paper lies within:

(1) The technical and economic indicators’ system of quality for metal-cutting tools taking into account uncertainty of the information for certain production conditions was created;
(2) The method for determining the adhesive component for the cutting friction coefficient in terms of use as contact surfaces those of the cylinder made of the processed material, and as target surfaces, directly applied surfaces of the tool blades were improved, which allows avoiding the destruction of the tool cutting part, as well as determining the frictional characteristics of the interaction of the processed material with the purchased tool, including with an unknown coating;
(3) Tools for quantitative assessment of the turning inserts quality based on simulation of the cutting process, considering the tribological interaction of tool and machined materials, and the tool edge radius were developed and implemented practically;
(4) The method for determining the intensity of cutting tool wear by cutting blade analysis with an electron microscope, an interferometer, and a dynamometer to determine the change in forces under machining was developed.

## 2. Research Methodology

### 2.1. A General Model for Choosing an Edge Cutting Tool for Optimal Cutting Conditions

**Figure 2:**
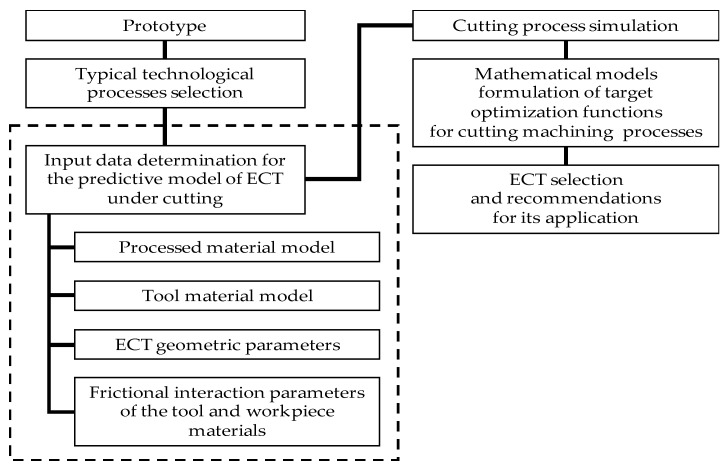
A generalized model of the edge cutting tools choice to ensure optimal cutting conditions.

One way to solve the issue of using IoT for the relationship between the cyber-physical system, the customer, and smart manufacturing is to virtualize the last work. Figure 2 presents a generalized *ECT* choice model to ensure optimal cutting conditions. The model basis is selecting a specific *ECT* using technical recommendations to exploit a particular “smart” production. A cyber-physical system should choose this scheme based on determining the machining process optimization’s objective function using these processes simulation. The optimization objective should consider analyzing the external and internal “smart” production context and customer requirements.

A modern approach to solving the problem’s optimal choice is based on operations research methods. According to this approach, the statement of optimizing cutting conditions problem consists of the fact that these requirements for the manufactured part and the known parameters of the machine-tool-part system need to find the parameters of the cutting process that provide extreme values of the optimization criteria for the technological process, in particular: technological cost, USD ($C^{\min}\to \min$); tool productivity, mm^2^/min (${П}_{F}\to \max$); durability period, min ($T^{cut}\to \max$). It leads to contextual quality management of the *ECT*’s operation.

The article proposes a complex indicator of the *ECT*’s quality, determined by the formula:

(1) $$Q^{ECT}=\sum_{i=1}^{N}B_{i}\cdot Q_{i},$$

where *Q_i_*—the relative quality indicator of *ECT* defining the ratio of the critical value of the optimality criterion of the technological process to the base value; *B_i_*—the value of the weight factor of the optimality criteria of the technological process.

*ECT*s with a minimum value of the technological cost, used at enterprises with specific machining modes for the related materials, are proposed as an essential indicator for determining the criterion of optimality for the technological process. The value of the weight factor of the technological process’s optimality criteria is due to determine with an expert evaluation providing that $\overset{N}{\underset{i=1}{\sum }}B_{i}=1$.

Two components are proposed as variable and constant parts to determine optimization target functions for the technological process, respectively, which depend on and are independent of cutting conditions. During the choice of *ECT*s for all assessment objects, the following values are considered: *T^cut^* = const—the depths of cut, mm; *D* = const—diameter of the workpiece, mm; *l* = const—the estimated length of the cutting stroke in the direction of the feed movement, mm. The mathematical model of situational management system [24] for the cutting process takes the following form:

(2) $$C^{\min}=C^{\mathrm{const}}/A^{\mathrm{var}};\;\;{П}_{F}=10^{-3}\cdot T^{\mathrm{cut}}\cdot A^{\mathrm{var}}$$

(3) $$\begin{array}{c} C^{\mathrm{const}}=\left[\left(\pi \cdot D\cdot l\right)/1000\right]\times \left(1/T^{cut}\right)\times [T^{cut}\cdot \left(E_{\mathrm{mach}}+E_{\mathrm{oper}}\right)+T_{\mathrm{sub}}\cdot (1+E_{\mathrm{oper}})\\ +K_{\mathrm{loss}}\cdot \left(C_{t}-C_{w}\right)/\left(j_{r}+1\right)];\\ H_{e}^{const}=M_{H}\cdot K_{pt}\cdot C^{en}\cdot l\cdot \pi \cdot D/1000;\;\;\;\;\;\;\;\;\;\;\;\;\;\;\;\;\;\;A^{\mathrm{var}}=V^{\mathrm{cut}}\cdot S^{\mathrm{cut}} \end{array}$$

where $T_{o}$—the main technological time, min; $E_{\mathrm{mach}},\;E_{\mathrm{oper}}$—the unit cost (per one minute of work of the machine and its operator), respectively, USD; $T_{\mathrm{sub}}$—machine downtime due to tool change, min; $C_{i}$—expenses, USD, associated with the operation of the tool according to its durable period $T^{cut}$, min; $K_{\mathrm{loss}}$—coefficient of the accidental loss of the tool (for polyhedral non-regrindable turning inserts $K_{\mathrm{loss}}=1$); $C_{t},\;C_{w}$—the initial cost of the tool and the cost of tool waste, USD; $j_{r}$—the number of regrinds allowed by the tool until full depreciation ($n_{r}=j_{r}+1$—the number of working faces of polyhedral non-regrindable turning inserts).

The mathematical formulation of determining the critical values of the technological process optimality criteria takes the following form (4):

(4) $$\left\{ \begin{array}{l} C^{\min}=f(t^{\mathrm{cut}};\;S^{\mathrm{cut}};\;V^{\mathrm{cut}})\to \min\\ {П}_{F}=f(t^{\mathrm{cut}};\;S^{\mathrm{cut}};\;V^{\mathrm{cut}};\;T^{\mathrm{cut}})\to \max\\ T^{\mathrm{cut}}=f(t^{\mathrm{cut}};\;S^{\mathrm{cut}};\;V^{\mathrm{cut}};\;\rho ;\;H_{excr})\geq T_{\mathrm{ac}}^{\mathrm{cut}}\\ R_{a}=0,2\cdot R_{z}=f(S^{\mathrm{cut}};\;\varphi ;\;\varphi_{1})\leq R_{a}^{ac} \end{array}\right.,$$

where $T^{\mathrm{cut}}$—time that characterizes the complex of properties associated with indicators of reliability and durability; $R_{z}$—the roughness of the part’s surface after machining (µm) that characterizes the property complex of the *ECT* associated with the indicators of the product purpose.

### 2.2. Simulation of Material Machining by Edge Cutting Tools

The solution to the problem mentioned above is realized using simulations, which were carried out during the research work “Increasing the vibration resource of the milling and turning processes of complex-profile parts made of difficult-to-machine materials based on the control and optimization of the geometry of the tools cutting part” (state registration No. 0113U000136, Ukraine), where authors were contractors. A finite element model of the cutting process is proposed, implemented based on the LS-DYNA solver with additional procedures in the form of the OCFEM module, which adapts the universal nonlinear dynamic solver to solve the problem modeling orthogonal free cutting. The main theoretical provisions from the mathematical apparatus used in the model are described in [25,26,27]. The finite element discretization of the workpiece and tool is performed by the four-node plane finite element (FE). Mesh generation is carried out by the Q-morph (advanced front) method [28] according to the given FE size on the body’s external contour. A set of geometric primitives defines the contour while generating a new mesh. Remeshing is based on FE edges of the base FE mesh. The FE model of the cutting process uses the mathematical theory of plasticity. One of the essential assumptions is the assumption of the isotropic incompressible continuum. Moreover, the material’s behavior during deformation is described by the yield surface’s behavior as the boundary between the elastic and plastic zones in each body point’s nine-dimensional stress space.

The result is a simulation model of the cutting process that considers the tool’s tribological interaction, workpiece materials, and the tool edge radius based on the adhesive component determination of the friction coefficient during cutting.

### 2.3. Method for Determining the Adhesion Component of the Average Friction Coefficient

**Figure 3:**
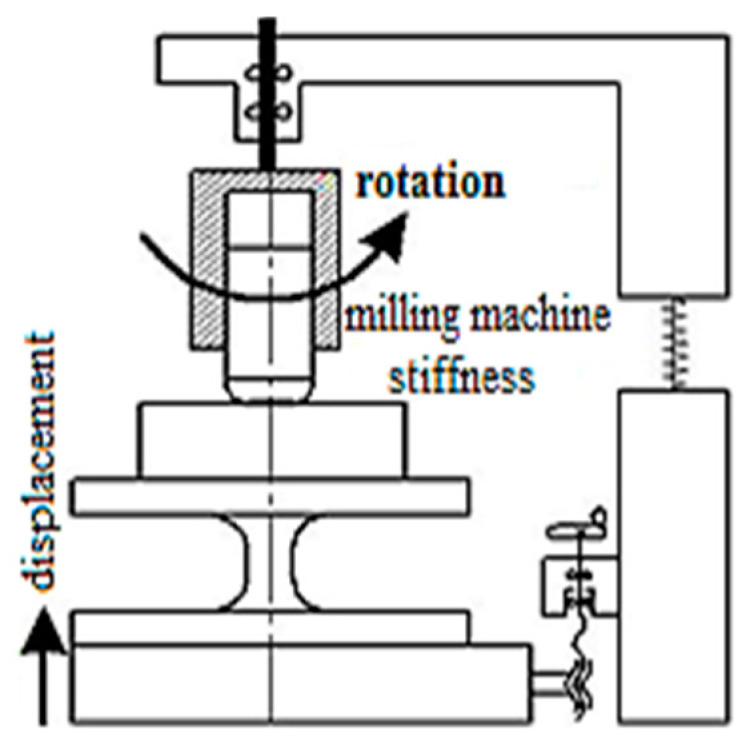
Loading schematics (new).

**Figure 4:**
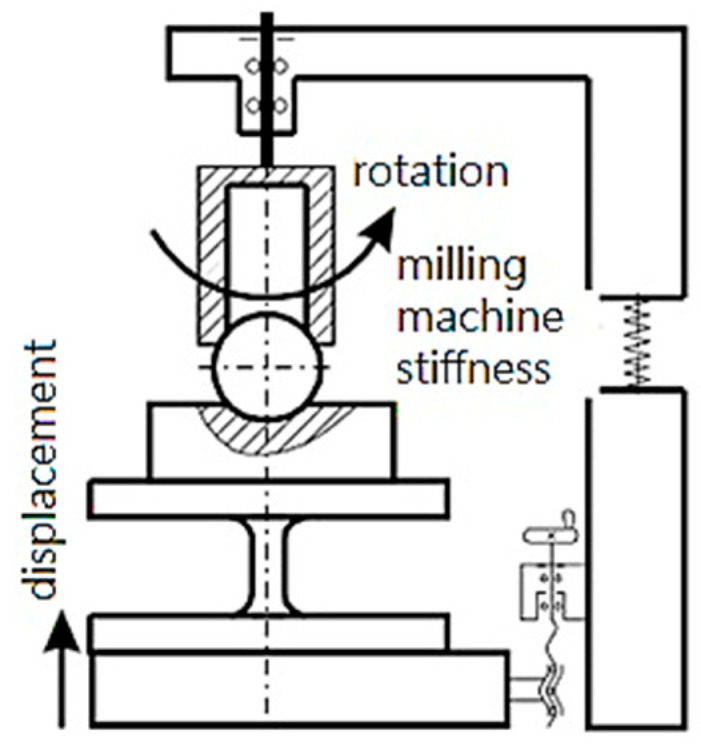
Loading schematics (old).

The authors in the study [29] propose an experimental method to determine the friction coefficient’s adhesive component [30] during cutting (Figure 3). This technique is based on the scheme proposed by D. Krivoruchko in [31] (Figure 4).

**Figure 5:**
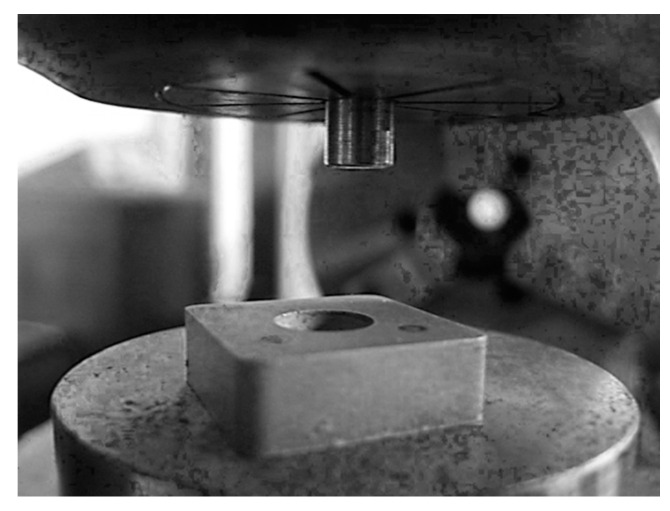
The device.

Figure 5 shows a part of a device for determining the adhesion component of the friction coefficient undercutting. The peculiarity of this scheme is, due to high pressures, a stagnant zone with a sufficiently high strength “attaches” both to the surface of the target surface and the contact surface of the indenter, especially close to microcavities filling the sample material. Suppose these bonds’ strength is higher than the stagnant zone’s material strength, then during the sample and indenter’s relative movement. In that case, destruction occurs along the stagnant zone, as a result of which the area of adhesive contact with internal friction (adhesive component) decreases, and the fraction of areas where external friction takes place (deformation component) grows.

### 2.4. Method for Determining the Surface Roughness of a Part after Machining

The determination of the surface roughness of a part after machining is performed according to the indicator $R_{z}^{eq}$, which is equivalent to the surface roughness of a part after machining, considering the smoothing depth $R_{p}$:

(5) $$R_{a}=0.2\cdot R_{z}^{eq},\;\;\;R_{z}^{eq}=h\cdot (1-t_{mA})+R_{p}\cdot t_{mA}$$

(6) $$t_{mA}=\left(\sum_{j}A^{\left(j\right)\;I}+\sum_{j}A^{\left(j\right)\;II}\right)/\left(\pi \cdot {R_{\max}}^{2}\right)$$

where *t_mA_*—the dimensionless coefficient of the specific filling of the area of the relative supporting surface of the adhesive bond at the height of the middle surface to the entire area of the machined surface. The areas’ values $\sum_{j}A^{\left(j\right)\;I},\;\sum_{j}A^{\left(j\right)\;II}$ $R_{p}$ are determined experimentally from the indentation image of the indenter onto the turning insert.

Variable h (theoretical surface roughness (µm)) is a criterion to be simulated. The criterion h is calculated according to generally known ratios depending on the edge geometry (e.g., for turning processes $h=1000\cdot S^{2}/(8\cdot \rho )$). The processed material model is specified as an empirical equation in the Johnson–Cook form [32]. According to the simulation results, the actual stress on the contact surface $\sigma_{nr}$ and the workpiece surface’s actual yield stress $\sigma_{s}$ is obtained.

### 2.5. Influence of the Tool Edge Radius on the Cutting Process Performance

The paper considers several assumptions. Firstly, the shape of turning inserts and their fixing methods were preliminarily defined according to terms of reference for processing system rigidity requirements.

Secondly, an influence of cutting tool edges radius value on the index of stability *ECT* in the absence of build-up forming according to the system of equations:

(7) $$\{\begin{array}{c} T^{\mathrm{cut}}\geq T_{\mathrm{ac}}^{\mathrm{cut}}\\ \rho_{\min}\leq \rho \leq \rho_{\max} \end{array}$$

It is established that there is no methodology for determining the intensity of cutting tool wear, which could be applied in machining and production practice. A number of assumptions are made in almost all known techniques, which significantly distort the actual picture of the interaction of the tool edge with the cut layer, chips, and surfaces on the workpiece.

The methodology for experimental wear rate determination is proposed for a cutting tool by analyzing a cutting edge. An electron microscope, an interferometer, and a dynamometer are used to determine cutting forces. This technique involves a full-scale cutting experiment. When stopping the cutting process, the tool wear is recorded according to several parameters: measurement of the tool edge radius ρ and the shape of the cutting edge and linear measurements of the tool wear area on the flank surface h_f_. The change in the shape and tool edge radius is controlled using an interferometer. At the initial moment of tool operation (during the running-in period), the experiment must be stopped, and the cutting edge checked every 3 s so as not to miss the end-of-life point and the beginning of the steady wear section. Then, in the area of constant blade wear, this period can be increased to 8–10 s. However, it is also essential to determine the point of the beginning of critical wear.

Next, a two-dimensional or three-dimensional image of the cutting edge of the insert is created. It makes it possible to measure the tool edge radius in the given (accepted) sections. For measuring the wearing area on the flank surface, it is necessary to take a photograph of the back surface of the turning insert using an electron microscope and combine it with a photograph of the scale bar, made using the same lens with the same lens magnification.

## 3. Results and Discussion

The practical implementation of scientific developments involved in real industrial production is given in Table 1. In the case of stability, the value does not correspond to the permissible value for each iteration while modeling the cutting process; the limiting values were changed by 5%. The optimal turning insert is No. 3 according to the criterion of technological cost in Table 1 and No. 1 by the tool productivity criterion.

The machined material is DIN C45 Steel; the base values correspond to the technological cost of turning insert No. 1, which was already used in the enterprise with specific machining modes for the related material.

**Figure 6:**
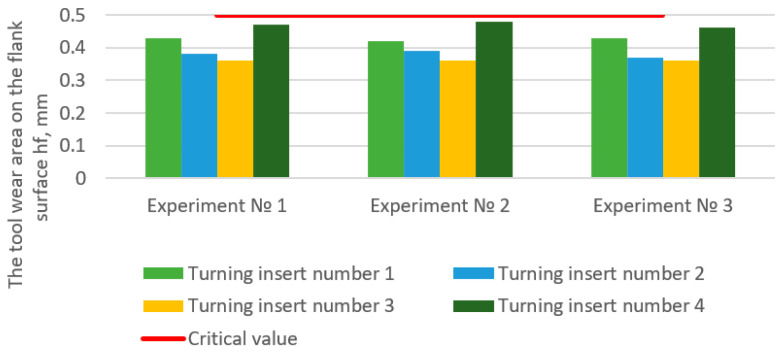
Results of a full-scale experiment with machining turning.

Figure 6 shows the results of a physical experiment with *ECT* rotation to determine the tool wear area on the flank surface *h_f_*. The optimal values were chosen as processing parameters for each insert according to the simulation results (“***” in Table 1). It is stated that the manufacturers’ recommended time to operate on a single working edge per turning insert is equal to 60 min. It was found that the recommended optimal machining modes that are identified during simulation do not exceed the established criterion for tool wear on the flank surface.

**Figure 7:**
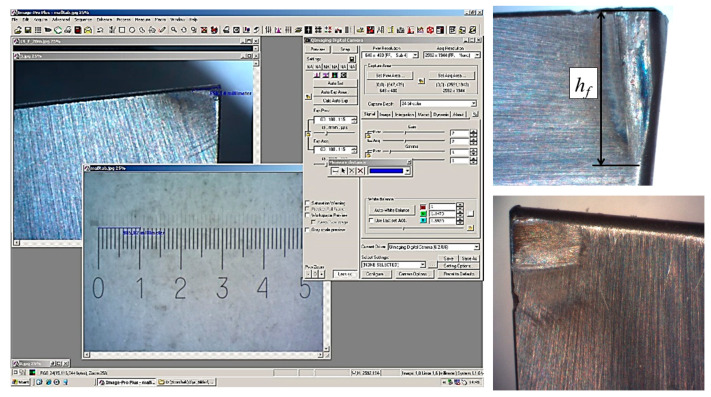
Photo of the data processing process results of a full-scale experiment with machining turning.

Machining was performed on a machine HAAS TL-1 using material according to the data from Table 1. Data processing process results of a full-scale experiment with machining turning are present in Figure 7.

Therefore, the suggested generalized *ECT* choice model to ensure optimal cutting conditions is one of the ways to solve the issue of using IoT for the relationship between the cyber-physical system, the customer, and “smart” production and allows one to implement the principle of production process virtualization.

The solid model implementation requires *ECT* manufacturers reorientation to the installation and standardization of data on tool parameters, in particular:

− The adhesion component of the friction coefficient undercutting for a specific pair of machining and tool materials (or coating of tool material);
− The influence of the tool edge radius on the performance of the cutting process of a particular material.

The implementation of simulation modeling requires the standardization of machining and instrumental materials models, open access to them, and transparent procedures for their verification.

There is a further need to improve the software for simulation modeling of cutting processes, mainly introducing a program module with automatic obtaining of target functions to optimize machining processes and increase accuracy and optimize the modeling process itself. The authors carried out four computer simulations of the cutting process to determine the target optimization functions of the machining modes for one turning insert (the time spent on the simulation of cutting was 64 h, computer parameters: processor—Intel^(R)^ Core^(TM)^ i5-6400 CPU @ 2.70GHz; 24 GB RAM).

Thus, using the proposed generalized *ECT* choice model to ensure optimal cutting conditions based on the developed tools for the material cutting process simulation involves Cloud Computing and Big Data [32,33].

## 4. Conclusions

The article aims to virtualize material machining (e.g., precision machining of *ECT*) by developing a general selecting *ECT* model to ensure optimal cutting conditions by using simulation and developing the technical and economic indicators system of the quality of the cutting tools.

A methodology for tools selection was presented in the paper. The proposed approach allows assigning optimal cutting parameters under specific production conditions. As a result, the technological process optimality criteria were obtained, and the corresponding extreme values of operational parameters were determined.

The impacts of the cutting-edge radius on cutting efficiency were identified for a particular material. Therefore, the proposed methodology can be realized by creating a standardized database to ensure quality technical and economic indicators for a metal-cutting tool. It allows substantiating a method for determining the adhesive component for the cutting friction coefficient in terms of use as contact surfaces were proposed.

The method for determining the intensity of cutting tool wear by cutting blade analysis was developed to determine the change in forces under machining. Tools for quantitative assessment of the turning inserts quality based on simulation of the cutting process, considering the tribological interaction of tool and machined materials, and the tool edge radius were developed and implemented practically.

During the production test results, the value of the technological cost of machining did not exceed 10% of the estimated value obtained by the actual machining modes at an enterprise. Moreover, due to the assumptions and definitions about numerical values of the simulation model, deviations of the tool wear did not exceed 15%.

Introducing the proposed model into machining practice can be realized by changing the business model of modern industrial production and the complete transformation of the modern model to introduce new non-food products from idea to final product.

## Figures and Tables

**Table 1:** Results of the *ECT* quality assessing and machining modes determination.

Turning Insert Number	Recommended *				Simulation Results **				Optimality Simulations Results ***			Optimality Criteria ****	
	V_max_, m/min	V_min_, m/min	S_max_, mm/rev	S_min_, mm/rev	V_max_, m/min	V_min_, m/min	S_max_, mm/rev	S_min_, mm/rev	t, mm	S_V_, mm/rev	V_S_, m/min	C^min^, USD	∏^F^, mm^2^/min
1	460	365	0.25	0.10	456	345	0.20	0.07	0.6	0.20	395	93.35	74.8
2	445	435	0.25	0.15	423	413	0.20	0.12	0.6	0.25	435	12.13	62.3
3	525	440	0.20	0.10	446	374	0.15	0.07	0.6	0.15	465	201.4	43.5
4	368	198	0.50	0.18	332	178	0.35	0.15	0.6	0.20	283	115.8	63.0
Base value												93.35	74.8

* Manufacturer-recommended value limits of machining modes for the respective turning inserts; ** obtained value limits of machining modes for the corresponding inserts based on the machining process simulation results; *** optimal processing parameters obtained by modeling the cutting process; **** the optimal values of the processing modes for the respective inserts according to the machining process simulation results according to the criterion of the minimum process expenses.

## Data Availability

Not applicable.
